# Plutonium (IV) Quantification in Technologically Relevant Media Using Potentiometric Sensor Array

**DOI:** 10.3390/s20061604

**Published:** 2020-03-13

**Authors:** Julia Savosina, Marina Agafonova-Moroz, Irina Yaroshenko, Julia Ashina, Vasily Babain, Alexander Lumpov, Andrey Legin, Dmitry Kirsanov

**Affiliations:** 1 Institute of Chemistry, St. Petersburg State University, Peterhof, Universitetsky prospect, 26, 198504 Saint-Petersburg, Russia; july-s@khlopin.ru (J.S.); ma-m@khlopin.ru (M.A.-M.); irina.s.yaroshenko@gmail.com (I.Y.); babainv@mail.ru (V.B.); lumpov@khlopin.ru (A.L.); andrey.legin@gmail.com (A.L.); 2 Khlopin Radium Institute, 2 Murinsky prospect, 28, 194021 Saint-Petersburg, Russia; 3 Laboratory of Artificial Sensory Systems, ITMO University, Kronverksky prospect, 49, 197101 Saint-Petersburg, Russia; ashina.julia91@gmail.com

**Keywords:** plutonium, potentiometric sensors, spent nuclear fuel, sensor array, PUREX

## Abstract

The quantification of plutonium in technological streams during spent nuclear fuel (SNF) reprocessing is an important practical task that has to be solved to ensure the safety of the process. Currently applied methods are tedious, time-consuming and can hardly be implemented in on-line mode. A fast and simple quantitative plutonium (IV) analysis using a potentiometric sensor array based on extracting agents is suggested in this study. The response of the set of specially designed PVC-plasticized membrane sensors can be related to plutonium content in solutions simulating real SNF-reprocessing media through multivariate regression modeling, providing 30% higher precision of plutonium quantification than optical spectroscopy.

## 1. Introduction

The typical industrial procedure for spent nuclear fuel (SNF) reprocessing implemented worldwide with minor variations is PUREX (Plutonium–Uranium Extraction)—a hydrometallurgical process where SNF is dissolved in concentrated nitric acid and the fuel components are separated from each other through the series of extraction/back-extraction steps between aqueous and organic phase to obtain uranium and plutonium [1]. Plutonium is one of the key target components and the content of this element must be thoroughly controlled through all the steps to ensure the safety and efficiency of the process. From the analytical chemistry point of view, the SNF reprocessing media is quite a challenging analyte due to its high radioactivity and strong acidity. In order to decrease the radiation burden for personnel and to ensure appropriate functioning of the analytical instrumentation, the samples taken from PUREX are normally diluted multiple times. The methods currently in use for plutonium quantification in process streams are mainly inductively coupled plasma atomic emission spectrometry (ICP-AES) [2] and mass spectrometry (ICP-MS) [3]. While providing perfect sensitivity and selectivity, these methods are rather time-consuming and can hardly be implemented in on-line mode. The results of such analyses are normally available only several hours after sampling, making timely interventions into the process run impossible. At some stages of the PUREX process, radiometric methods such as alpha spectrometry are also applied for plutonium quantification, but they require sample preparation as well.

In recent years, a novel approach towards analysis of PUREX process streams has emerged. It is based on the use of modern fiber optic probe UV-Vis-NIR and Raman spectrometers combined with chemometric data processing [4,5,6,7,8]. It was shown to be applicable for on-line determination of uranium, plutonium, nitric acid and lanthanides [9,10,11,12,13]. However, optical spectroscopy tools suffer from the inability to deal with non-transparent and highly scattering media, which is often the case at certain stages of PUREX. Moreover, in some cases optical spectroscopy cannot ensure sufficiently low detection limits for plutonium. In this respect, electrochemical sensing appears to be an attractive addition to these methods, as it can provide the quantification of very low concentrations of target analytes and allows measuring in turbid liquids.

Electrochemical sensing of plutonium is rather poorly covered in literature. There are several reports describing voltammetric sensors for plutonium [14,15,16,17,18] based on glassy carbon, Pt and Au working electrodes. In [15], the use of a glassy carbon electrode for plutonium determination in 0.5 M perchloric acid was suggested. The working concentration range in this study was 2.24 × 10 ^−4^–4.48 × 10^−3^ mol/L. Although direct voltammetric sensing of plutonium seems viable, it has certain drawbacks, like poisoning of the Pt and Au working electrode surfaces through the formation of Pt-O and Au-O bonds and the inferior sensing properties of glassy carbon electrodes. A number of studies were further devoted to modification of electrode surfaces [16,17,18]. In [17], the authors have modified gold electrodes with single-walled carbon nanotubes for simultaneous determination of plutonium and uranium. This configuration allowed the determination of uranium and plutonium in 1 M sulfuric acid. The detection limit was 8.2 mM for plutonium and 2.4 mM for uranium. Then the proposed electrode was tested on fuel samples for Fast Breeder Test Reactor (FBTR) and has shown acceptable analytical performance. The modification of glassy carbon electrode surfaces with ruthenium nanoparticles [18] allowed simultaneous quantification of Pu and Np with detection limits of 1.5 and 6.5 µmol/L correspondingly.

Potentiometric sensing of plutonium is much more challenging, and we have found only a single attempt to create such sensors [19]. Besides radioactivity issues, this is due to the fact that the chemistry of neighboring actinides is very similar and ligands providing the binding of plutonium will normally bind uranium as well. Thus, the selectivity of plasticized polymeric membranes based on such ligands will be insufficient for real application in SNF analysis. Another serious concern is the low pH level of the target samples contributing to ligand protonation and deteriorating sensitivity towards plutonium. All these issues are discussed in [19].

It was shown that the insufficient potentiometric selectivity of discrete sensors can be effectively compensated by the application of sensor arrays with varying cross-sensitivity patterns. The mixtures of lanthanides with very similar properties were effectively resolved using the set of potentiometric sensors based on various ligands suggested for lanthanide and actinide separation [20,21]. In this study, the extension of this methodology towards plutonium analysis is suggested. The quantification of plutonium in PUREX model solutions with potentiometric multisensor array is reported for the first time.

## 2. Materials and Methods

### 2.1. Samples

Individual aqueous solutions of plutonium were prepared from PuO_2_ and HNO_3_ according to [14]. The stock solution was purified from daughter products at an ion-exchange column [14]. The concentration of plutonium was determined by radiometric method. The Pu^4+^ form was stable at these conditions. It must be pointed out that the major isotope is ^239^Pu, which has a specific activity of about 2.3 GBq/g and thus all standard precautions for handling radioactive solutions were carefully observed during the experiment. Aqueous solutions of uranyl nitrate were prepared by dissolving (UO_2_(NO_3_)_2_·6H_2_O, supplied by JSC Isotope, Moscow, Russia) in distilled water. Then the concentration of uranium was determined by the titration of uranium with ammonium vanadate (barium diphenylamine sulfonate as indicator) [22]. The concentration of nitric acid was determined by titration with 0.1 M NaOH (methyl orange as indicator).

Three sets of plutonium solutions were studied. The first one contained 1 × 10^−7^–1 × 10^−4^ M plutonium(IV) in 1.5 M nitric acid and was employed to study the sensitivity of the sensors in nitric acid. The second set was employed to explore the sensitivity of the sensors to Pu^4+^ in the presence of technologically relevant uranium concentrations. This set contained 10–50 mg/L Pu^4+^ in 1.5 M HNO_3_ and 500 mg/L of uranium. The third set was employed as a simulation of the real industrial PUREX conditions. It contained 20 mixtures of Pu (10–50 mg/L) and U (10–1000 mg/L) in 1.5 M nitric acid, closely mimicking the composition of the first PUREX cycle raffinate. Compositions of these mixtures are given in Table 1.

Although there are special calibration designs for the two component mixtures described in literature [23,24], in this case we had to deal with the limited number of fixed concentration levels to meet safety requirements of Pu^4+^ solutions handling and to minimize the waste amount.

### 2.2. Sensor Preparation and Potentiometric Measurements

The sensor array consisted of 17 potentiometric polymeric membrane electrodes. Various ligands suggested earlier in liquid extraction processes for the separation and concentration of actinides and lanthanides were employed as membrane active compounds. The choice of particular extracting agents providing for the diversity of sensor response patterns was made from the literature. Such an approach was recently found to be applicable for the quantification of thorium [25]. The details of sensor membrane compositions and preparation protocol are provided in the Supplementary Materials Table S1. Figure S1 (Supplementary Materials) shows the visual appearance of the developed sensor array.

The potentiometric measurements were carried out in a 25 mL Teflon beaker under stirring using the galvanic cell:

Cu | Ag | AgCl, KCl_sat_ | sample solution | sensor membrane | 0.01 M NaCl, Ag | AgCl | Cu.

The standard Ag/AgCl electrode EVL 1M 3.1 (ZIP, Gomel, Belorussia) was used as a reference electrode. The glass electrode ESL 63-07 (ZIP, Gomel, Belorussia) was employed to monitor the pH of the samples; it was calibrated with standard buffer solutions prior to the experiment. Potential values were measured with ± 0.1 mV precision using a 20-channel digital mV-meter (Sensor Systems, LLC, St. Petersburg, Russia) with high input impedance, connected to a PC for data acquisition. Sensor readings were registered after 3 min measurements in each studied solution.

All numerical values presented below were averaged over three replicated measurements.

Potentiometric sensitivity of the sensors was determined as the slope of the linear part of the sensor response curve in lg*C* − E (mV) coordinates, where *C* is the primary ion concentration in the sample. All the potentiometric measurements were performed in 1.5 M HNO_3_ (pH ≈ 0) to suppress hydrolysis and to provide constant ion strength. Due to this, we used concentration values throughout the experiment and did not perform the correction for activity. Moreover, highly acidic conditions are very typical for PUREX.

The determination of selectivity coefficients for plutonium was performed by the separate solution method (SSM) in 1 × 10^−4^ M solutions according to the equation:

(1) $$\mathrm{lg}K_{Pu/X}=\frac{E_{X}-E_{Pu}}{Sensitivity}+\mathrm{lg}\left(\frac{10^{-4}}{{\left(10^{-4}\right)}^{4/z_{X}}}\right),$$

where K*_Pu/X_* is the selectivity coefficient (Pu—primary ion, X—interfering ion); *E_Pu_* and *E_x_* are EMF values of the cell upon the measurements in monocomponent solutions of Pu and X, accordingly; and *Z_x_* is the charge of the interfering ion.

The selectivity coefficients were calculated against the following ions: UO_2_^2+^, Th^4+^, La^3+^, Ce^3+^, Pr^3+^, Nd^3+^, Sm^3+^, Eu^3+^, Gd^3+^—typical constituents of SNF reprocessing media.

### 2.3. UV-Vis-NIR Spectroscopic Measurements

In order to compare the performance of the sensors with that of known optical spectroscopy methods, the mixtures presented in Table 1 were also measured with UV-Vis-NIR spectrometer AvaSpec (Avantes B.V., Apeldoorn, The Netherlands) in the 184–1100 nm range with 0.53 nm step. The sample cell (10 mm × 10 mm × 40 mm, L × W × H) containing 0.5 mL of the sample was placed in a special holder for transmission measurements. The holder was coupled with two optical fibers supplying the light beam from a deuterium-halogen light source AvaLight-DH-S-BAL (Avantes B.V., Apeldoorn, The Netherlands) and receiving the transmitted light to convey it to the spectrophotometer. Proprietary Avantes software was used for data registration. The final spectrum of each particular sample was an average from over 150 repeated scans. All measurements were performed at room temperature.

### 2.4. Multivariate Data Processing

The multivariate data obtained from the sensor array during the measurements in mixtures of Pu and U and the data from the spectrophotometric experiment were processed using Projection on Latent Structure (PLS) regression [26]. All sensor responses were mean centered and scaled with the standard deviation prior to the modeling. Root mean square error of prediction (RMSEP) was used to evaluate models’ performance:

(2) $$RMSEP=\sqrt{\sum_{i=1}^{n}\frac{{\left(y_{i,ref}-y_{i,pred}\right)}^{2}}{n},}$$

where *y_i,ref_* and *y_i,pred_* are the reference and predicted concentrations of an analyte in the i*th* sample and n is a total number of samples in a test set.

All calculations were performed with The Unscrambler 9.7 software package (CAMO, Oslo, Norway).

## 3. Results

### 3.1. Sensitivity and Selectivity of the Individual Sensors

The following results of potentiometric sensitivity towards plutonium (IV) are the first ever data published on this topic.

**Figure 1:**
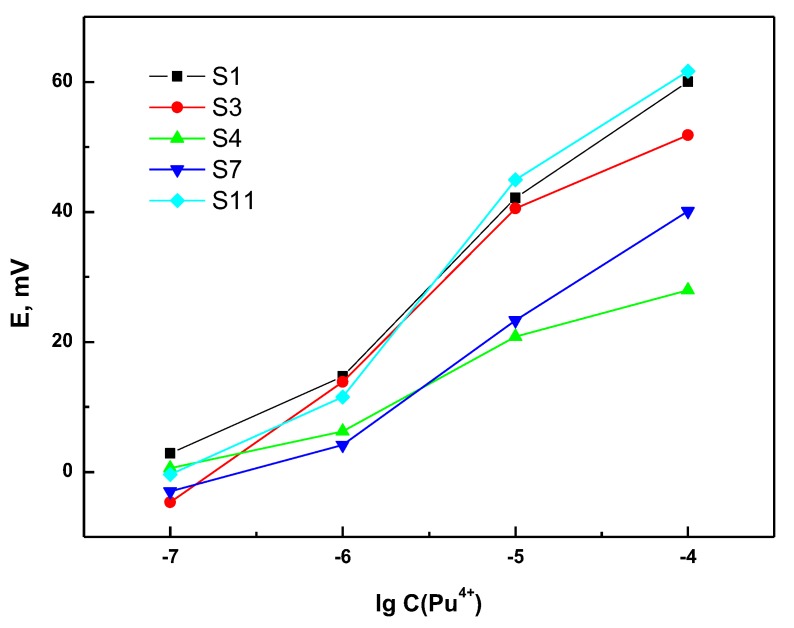
Typical potentiometric response curves in Pu^4+^ aqueous solutions with 1.5 M HNO_3_. The presented EMF values are determined with ± 0.1 mV precision.

**Figure 2:**
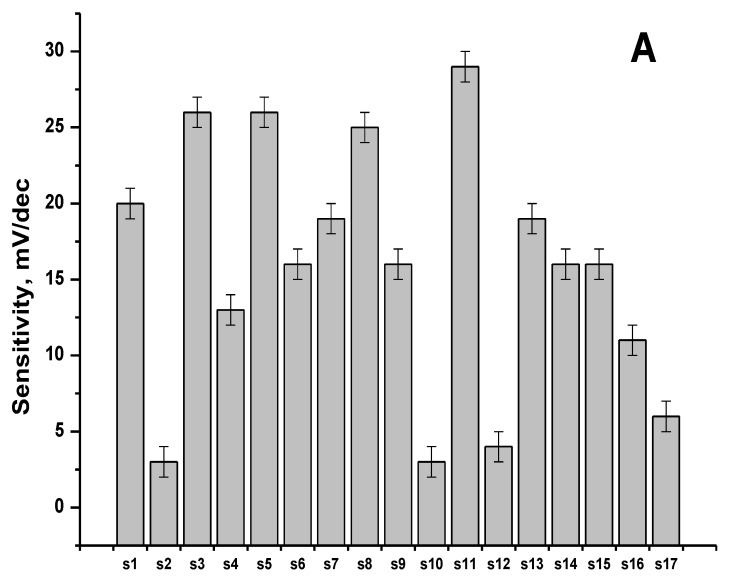

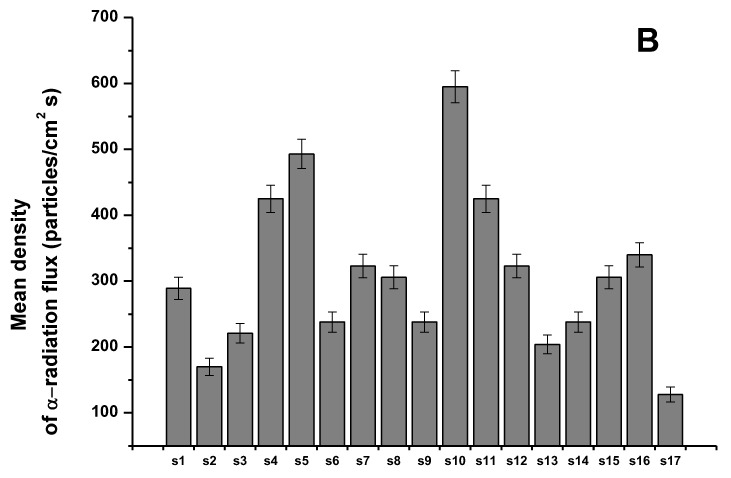
(**A**) Potentiometric sensitivity values to Pu^4+^ for the studied sensors. (**B**) Radioactivity of the sensor membranes after the measurements in plutonium solutions.

In order to study sensor sensitivity to Pu^4+^, measurements of 1 × 10^−7^–1 × 10^−4^ M plutonium in 1.5 M nitric acid were performed. Each sensor demonstrated stable potential readings after 10–20 s of immersion in a solution. The typical responses of several sensors are shown in Figure 1. The Nernstian value of sensitivity towards quadruply charged cations is 14.7 mV/dec. Figure 2A summarizes the Pu^4+^ sensitivity values for all studied sensors.

Due to personnel radiation exposure considerations, each separate experiment with radioactive plutonium solutions must be carefully justified. In order to minimize such exposure, no special measurements to assess the limits of detection (LD) for Pu^4+^ were performed. LDs were estimated from the data reported just above and they were around 5 × 10^−7^ M Pu^4+^ for most of the sensors.

After the first series of measurements in plutonium solutions, it was found that sensor membranes became radioactive. Using DKS-96 (SPC “Doza” Ltd., Zelenograd, Moscow, Russia), we have measured the average density of alpha-radiation flux for each of the sensors with 40 s exposition in three replicates (Figure 2B) at a distance of 1 cm away from the membrane surface. There was no correlation observed between the radioactivity values and the sensitivity values of the sensor membranes (compare the patterns in Figure 2A,B). Despite the high radioactivity of the materials, sensor responses were stable over the whole duration of the experiment (about 45 days).

Selectivity was evaluated for the sensors that demonstrated Pu^4+^ sensitivity values above 10 mV/dec. The sensor compositions S2, S10, S12, S17 were not considered. Selectivity data are summarized in Table S2 (Supplementary Materials). It is important to mention that thermodynamically strict consideration of these values is not possible, since the responses of the sensors towards the primary and interfering ions are often far from theoretical values. Moreover, due to the second term in Equation (1), the calculation of Pu^4+^/UO_2_^2+^ selectivity values (where this term equals 4) appears to be strongly shifted in the favor of uranium. Thus, the values in Table S2 should be taken with some skepticism. Even their qualitative value is questionable in the case of uranium, where all of the sensors appear to be selective towards UO_2_^2+^, which is in contradiction with further results of this study and relates to the nature of selectivity calculation formalism, where theoretical response values for all the studied ions are required.

### 3.2. Sensor Response towards Pu^4+^ in Presence of UO_2_^2+^

**Figure 3:**
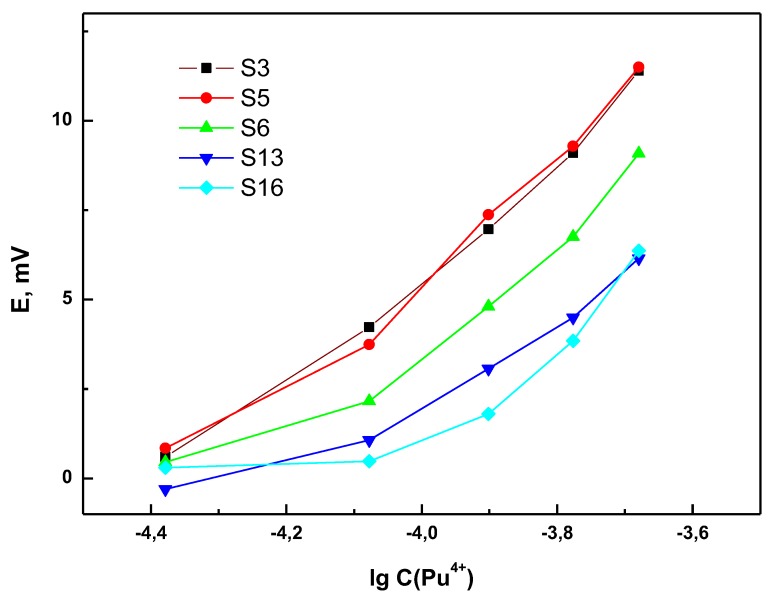
Potentiometric response curves to Pu^4+^ obtained in the presence of 500 mg/L U in 1.5 M HNO_3_.

Since the selectivity evaluated with SSM towards uranium was questionable (Table S2) but crucial for SNF related applications, a series of measurements of Pu solutions containing the constant background of U in PUREX relevant concentration levels was performed. Samples containing 10–50 mg/L of Pu were measured in mixtures with 500 mg/L U in 1.5 M HNO_3_. Typical response curves for Pu^4+^ in these conditions are presented in Figure 3. A significant deterioration of the sensitivity towards Pu in the presence of uranyl ions was observed. While the sensitivities in individual plutonium (IV) solutions varied from 5 to almost 30 mV/dec, in the presence of uranyl ions, these values are in the range of 0–20 mV/dec (see Figure S2 in Supplementary Materials). This is understood to happen because the chemistry of neighboring actinides is very similar and organic ligands providing the complexation of plutonium ions can also bind uranyl.

Nevertheless, in spite of the significant excess of UO_2_^2+^ over Pu^4+^ and apparent SSM selectivity values obtained before, we have still observed reasonably high sensitivity towards Pu^4+^ in some of the sensors.

### 3.3. Multisensor Measurements in Simulated PUREX Solutions

**Figure 4:**
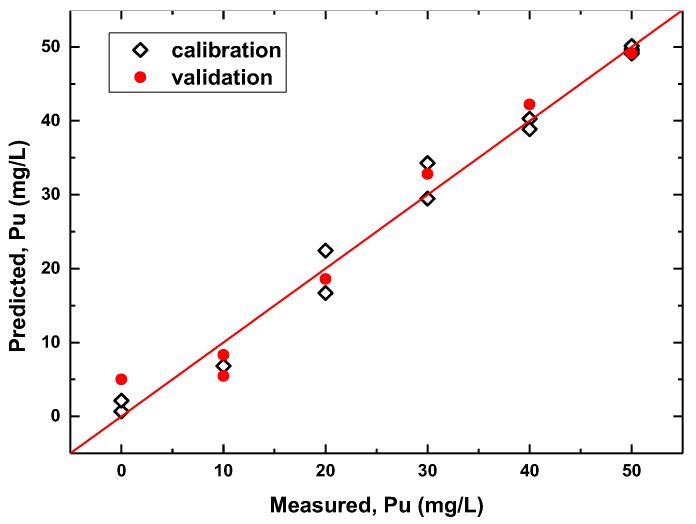
“Measured vs. predicted” plot for partial least squares regression (PLS) model based on potentiometric sensor data obtained in simulated PUREX solutions. Validation with 7 randomly chosen samples, 4 latent variables, R^2^ 0.98/0.94, RMSE 2.0/4.4 mg/L (calibration/validation).

In order to explore the performance of the potentiometric sensing approach in the quantification of Pu in technologically relevant media, measurements were performed with the sensor array of 13 electrodes (except S2, S10, S12 and S17) in the set of simulated PUREX solutions mimicking the composition of the first PUREX cycle raffinate (Table 1). It must be pointed out that in the presence of varying uranium content, the traditional univariate way of potentiometric data processing using the Nikolsky-Eisenman equation is hardly possible (see, e.g., Figure S3 (Supplementary Materials) to assess the sensors’ response shapes), since variations in uranium content contribute significantly to the potential readings of the sensors. Thus, in order to relate sensor response with plutonium concentration, the data from the sensor array were modeled using a multivariate approach. The sensor readings obtained in the mixtures were organized as *M* × *N* matrix, where *M* is the number of samples and *N* is the number of sensors. This matrix was used as *X* in linear regression equation *Y* = B*X*, where variable *Y* is the column vector of known analyte concentrations. Calculation of regression coefficients vector B was performed according to the PLS algorithm. The results of the modeling can be represented as “measured vs. predicted” plots. Figure 4 shows a plot of such a PLS model validated with seven randomly chosen samples. It can be seen that reliable plutonium quantification is possible. The number of samples was rather limited due to safety reasons. Thus, we applied a 20-fold random split cross-validation, each time using 13 samples for calibration and seven for validation, to get an estimate of the predictive performance of the sensor array. The average RMSEP for plutonium content prediction was 3.9 mg/L in the concentration range 0–50 mg/L. It was also possible to construct predictive PLS models for simultaneous determination of uranium content in these samples with an average RMSEP of 73 mg/L (concentration range 10–1000 mg/L) using the same 20-fold random split validation procedure.

The same samples were also measured with a UV-Vis-NIR spectrometer. The observed spectral shapes were similar to those reported in the literature [8]. The same 20-fold random split cross-validation procedure returned an average RMSEP of 6.1 mg/L of plutonium (compared to 3.9 mg/L by multisensor array). The models were built using the wavelength range 350–800 nm. Quantification of uranium (by spectroscopic method?) was possible with the average RMSEP value of 47 mg/L.

## 4. Discussion

In spite of the unusually high acidity for PVC membrane sensors (pH ≈ 0), it was still possible to determine Pu^4+^. This indicates that ligand protonation constants for most of the sensors are smaller than Pu^4+^(L)_n_ complex formation constants. Moreover, several membrane compositions provide super-Nernstian slope values. Another important issue is that certain similarities between extraction behavior of the ligands and their sensitivity towards plutonium were observed. For example, the sensors S3, S8 and S14 show rather high potentiometric sensitivity to Pu^4+^. These sensors are based on diphosphine dioxide (S3) and carbamoyl phosphine oxides (S8 and S14). These ligands are well known as powerful extracting agents for tetravalent actinides [27,28,29]. The sensors based on diamides of pyridine-2,6-dicarboxylic acid (S1, S5, S9, S11, S13) also demonstrate considerable sensitivity to plutonium, which is in agreement with the liquid extraction behavior of these ligands [30]. On the other hand, the sensors based on podands (S2 and S12) do not show a considerable response to Pu^4+^, and this corresponds well to the observations obtained in liquid extraction where only *N*,*N*-dialkyl amides extract Pu^4+^ effectively [31].

Selectivity of the sensors in the Pu^4+^/Th^4+^ pair is in the favor of Pu^4+^. In the presence of lanthanides, the selectivity varies depending on the particular sensor membrane composition. For some of the sensors, it slightly grows with the growth of atomic number from La to Yb (S7, S14, S15, S17). For some of the compositions, an opposite trend can be observed (e.g., S1, S6). In certain cases, the lanthanide selectivity values are distributed quite uniformly (e.g., S5, S8). The absence of sharp selectivity patterns in lanthanide series is very natural due to their similar electronic structure and chemical properties, despite the number of reports declaring the development of selective sensors towards individual lanthanides (see, e.g., [32,33,34,35,36]).

In the presence of high concentrations of uranium, we observed high Pu^4+^ sensitivity in the sensors. These results confirm the relevance of a multisensor approach instead of individual sensors for PUREX related analytical tasks.

Simulated PUREX raffinate solutions were key objects in this investigation. The potentiometric sensor array tested in raffinate solutions has shown reasonable analytical performance for quantitative determination of both plutonium and uranium. The RMSEP values for plutonium quantification are 30% lower than those achieved with optical spectroscopy in the same samples. The attained precision is sufficient for the purposes of technological monitoring.

## 5. Conclusions

In spite of the fact that plutonium quantification in PUREX process streams is a very important analytical task, there is a very limited number of papers devoted to the development of electrochemical sensors for plutonium. Selective quantification of plutonium in PUREX media is quite challenging due to the presence of other actinides and lanthanides that have rather similar chemical properties. In this study, we have suggested a simple and cost-effective potentiometric multisensor system to perform fast Pu^4+^ quantification in complex acidic samples simulating PUREX raffinate composition. It was found that the quantitative determination of plutonium is possible with the precision sufficient for technological monitoring needs, even with a large excess of uranium.

## Figures and Tables

**Table 1:** The composition of 20 Pu-U mixtures (mg/L) in 1.5 M HNO_3_.

**# Mixture**	**1**	**2**	**3**	**4**	**5**	**6**	**7**	**8**	**9**	**10**
Pu	0	0	0	10	10	10	20	20	20	30
U	10	400	1000	200	600	1000	10	400	800	200
**# Mixture**	**11**	**12**	**13**	**14**	**15**	**16**	**17**	**18**	**19**	**20**
Pu	30	30	40	40	40	50	50	50	50	50
U	600	1000	10	400	800	10	200	400	600	1000

## Supplementary Materials

The following are available online at https://www.mdpi.com/1424-8220/20/6/1604/s1, Table S1: List of sensors ligands; Table S2: lg K(Pu^4+^/Me*^n^*^+^) selectivity values of the sensors for selected actinides and lanthanides; Figure S1: Visual appearance of the developed sensor array; Figure S2: Sensitivity to Pu^4+^ in the presence of 500 mg/L uranium; Figure S3: Sensor responses in the simulated PUREX solutions.
