# Supplementary material for: Plutonium (IV) Quantification in Technologically Relevant Media Using Potentiometric Sensor Array
Source: Sensors (Basel). 2020 Mar 13;20(6):1604. doi: 10.3390/s20061604 (PMC7147468; doi:10.3390/s20061604)
Supplement: Supplementary file 1 [file sensors-20-01604-s001.pdf]

SUPPLEMENTARY MATERIAL

Plutonium (IV) Quantification in Technologically Relevant Media Using Potentiometric Sensor Array

Julia Savosina [1,2], Marina Agafonova-Moroz [1,2], Irina Yaroshenko [1,3], Julia Ashina [3], Vasily Babain [1], Alexander Lumpov [1,2], Andrey Legin [1,3] and Dmitry Kirsanov [1,3,*]

[1] Institute of Chemistry, St. Petersburg State University, Peterhof, Universitetsky prospect, 26, 198504 Saint-Petersburg, Russia; july-s@khlopin.ru (J.S.); ma-m@khlopin.ru (M.A.-M.); irina.s.yaroshenko@gmail.com (I.Y.); babainv@mail.ru (V.B.) ; lumpov@khlopin.ru (A.Lumpov); andrey.legin@gmail.com (A.Legin)

[2] Khlopin Radium Institute, 2 Murinsky prospect, 28, 194021 Saint-Petersburg, Russia

[3] Laboratory of Artificial Sensory Systems, ITMO University, Kronverksky prospect, 49, 197101 Saint-Petersburg, Russia; ashina.julia91@gmail.com

* Correspondence: d.kirsanov@gmail.com; Tel.: + 7-921-333-1246

Received: 5 February 2020; Accepted: 10 March 2020; Published: date

Sensor membrane preparation

The sensor membranes were prepared using a standard protocol. 17 ligands were selected for membrane preparation, based on liquid extraction literature data. The list of the ligands along with corresponding literature references describing their synthesis and extraction behavior is given in the Table S1. All ligands were kindly provided by Khlopin Radium Institute (St. Petersburg, Russia). The polymeric membrane matrix of electrodes was composed of poly(vinyl chloride) (PVC) (33 wt.%) and 2 - nitrophenyloctyl ether (NPOE) as a plasticizer (64–65 wt.%). Potassium tetrakis[3,5-bis(trifluorometyl)phenyl]borate (KTFPB) or the acidic form of chlorinated cobalt dicarbollide (CCD) were used as cation-exchangers (10 mmol/kg). PVC, NPOE and KTFPB were obtained from Merck (Darmstadt, Germany). CCD was kindly provided by Katchem (Prague, Czech Republic). All sensor membranes contained 50 mmol/kg of one of the ligands listed in the Table S1.

In order to prepare the membranes the weighted amounts of membrane components were dissolved in freshly distilled tetrahydrofuran and poured into flat bottomed Teflon beakers. The cocktails were left overnight for solvent evaporation. Three sensor membranes 4 mm in diameter were cut from the parent membrane of each composition and glued upon the end of PVC sensor bodies (6 mm in diameter) with PVC-cyclohexanone mixture. The sensor bodies were equipped with Ag/AgCl inner reference electrodes prepared from 5 mm long pieces of silver wire (0.5 mm in diameter) covered with AgCl layer in the electrochemical process.

Table S1. The components of the sensor membranes.

Nature of the ligand	Chemical structure	Cation exchanger	Sensor number	Ref.
Diamides of dipicolinic acid	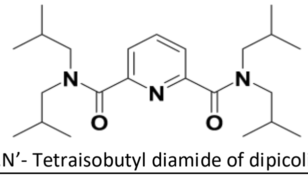 N,N',N,N'- Tetraisobutyl diamide of dipicolinic acid	KTFPB	S1	[s1]
	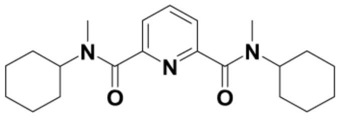 N,N'-Dimethyl-N,N'-dicyclo-hexyldiamide of dipicolinic acid	KTFPB	S5	[s2]
	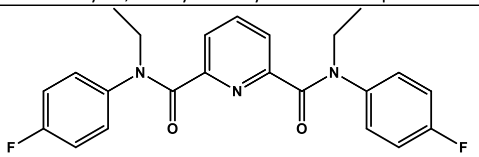 N,N'-Diethyl-N,N'-di(p-fluoro)phenyl diamide of dipicolinic acid	KTFPB	S11	[s2]

	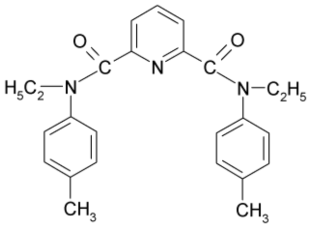 N, N′-Diethyl-N, N′-di-p-tolyldiamide of dipicolinic acid	KTFPB	S13	[s3]
Nitrogen containing oxa-nonanes	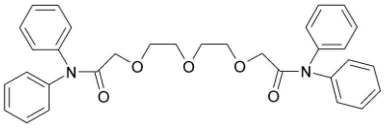 1,9-Bis-(diphenylcarbamoyl)-2,5,8-trioxanonane	CCD	S2	[s4]
	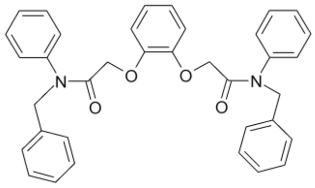 1,6-Bis-(benzylphenylcarbamoyl)-3-benzo-2,5-oxahexane	KTFPB	S12	[s4]
Phosphine oxides	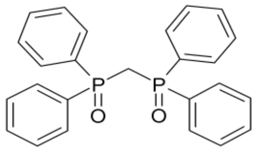 Tetraphenylmethylene diphosphine dioxide	KTFPB	S3	[s5]
	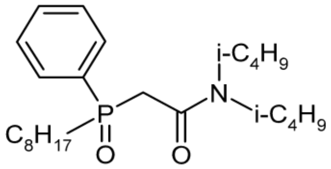 Phenyloctyl-N,N-di-i-butylcarbamoyl-methylen phosphineoxide	CCD	S8	[s6]
	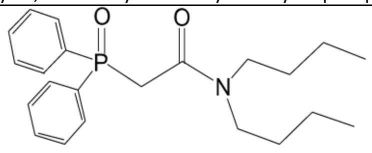 Diphenyl-N,N-di-n-butylcarbamoyl-methylphosphinoxide	CCD	S14	[s7]
Amides of organic acids	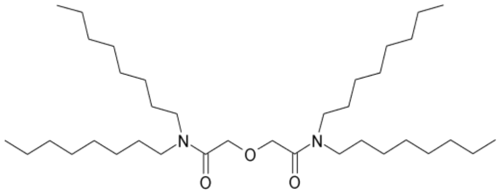 N,N,N′,N′-Tetraoctyldiamide of diglycolic acid	CCD	S4	[s8]
	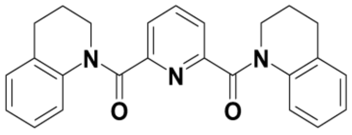 Pyridine-2,6-dicarboxylic acid bis(1,2,3,4-tetrahydroquinolide)	KTFPB	S9	[s1]
	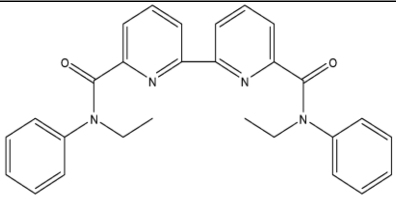 N,N′-diethyl-N,N′-diphenyldiamide of 2,2′-dipyridyl-6,6′-dicarboxylic acid	KTFPB	S10	[s9]

	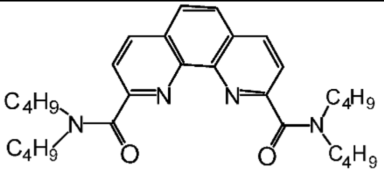 N^2,N^2,N^9,N^9-Tetrabutyl -1,10-phenanthroline-2,9-dicarboxamide	KTFPB	S17	[s7]
	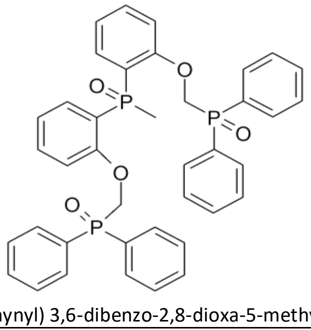 1,9-Bis-(diphenylphosphynyl) 3,6-dibenzo-2,8-dioxa-5-methyl-phosphineoxanonane	KTFPB	S6	[s10]
Phosphoryl containing oxa-nonanes	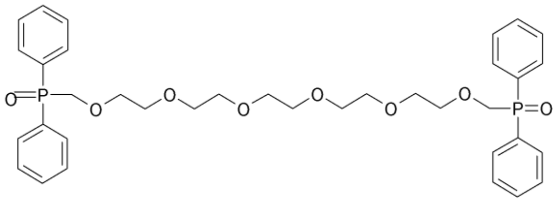 1,18-Bis-(diphenylphosphynyl)-2,5,8,11,14,17-hexaoxaoctadecane	CCD	S15	[s10]
	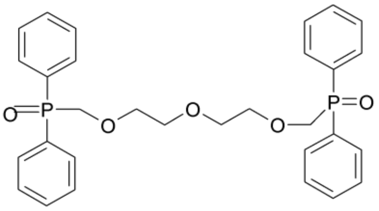 1,9-Bis-(diphenylphosphynyl)-2,5,8-trioxanonane	CCD	S16	[s10]
Calixarene	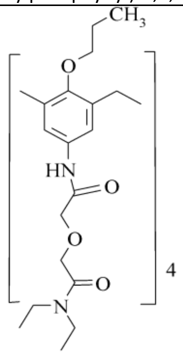 5,11,17,23-Tetra(diethylcarbamoyl-ethoxymethylcarboxamido)-25,26,27,28-tetrapropoxycalix[4]aren	CCD	S7	[s11]

Table S2. lg $K(Pu^{4+}/Me^{n+})$ selectivity values of the sensors for selected actinides and lanthanides.

	S1	S3	S4	S5	S6	S7	S8	S9	S11	S13	S14	S15	S16
**La**	-0.4	-0.1	0.7	-0.1	0.7	0.4	0.9	-2.4	-0.2	-0.6	-0.7	-1.2	-1.7
**Ce**	-0.4	-0.3	-0.6	0.0	0.4	0.0	0.4	-1.4	-0.1	-0.8	-0.5	-0.8	-1.4
**Pr**	0.2	0.0	0.3	0.4	0.4	0.1	0.6	-0.9	0.5	-0.4	-0.3	-0.3	-1.6
**Nd**	0.4	0.0	0.4	0.4	2.6	-2.0	1.1	-2.6	0.5	-0.5	-1.6	-1.4	-1.9
**Sm**	0.2	-0.1	0.4	0.3	1.8	-0.7	0.8	-2.6	-0.1	-0.8	-1.5	-1.4	-2.0
**Eu**	0.2	-0.5	0.0	-0.1	1.4	-0.5	0.6	-3.3	0.2	-0.8	-2.4	-2.0	-3.1
**Gd**	0.1	-0.5	-0.1	0.1	1.2	-0.8	0.6	-2.5	0.0	-0.8	-1.8	-1.5	-2.5
**Yb**	0.4	-0.3	0.1	0.0	1.3	-2.3	0.6	-3.7	0.3	-0.8	-3.0	-2.6	-3.2
**Th**	-0.9	-0.6	-0.9	-0.7	1.3	-1.3	0.1	-2.8	-2.1	-1.6	-2.0	-1.8	-2.3
**U**	3.1	3.3	3.5	2.2	2.4	3.7	2.5	3.7	2.3	2.8	5.9	6.2	4.1

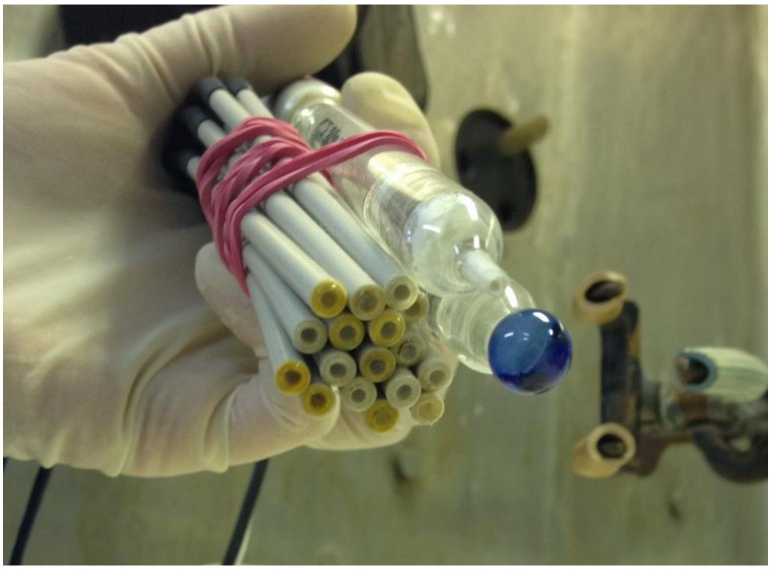

Figure S1. Visual appearance of the developed sensor array.

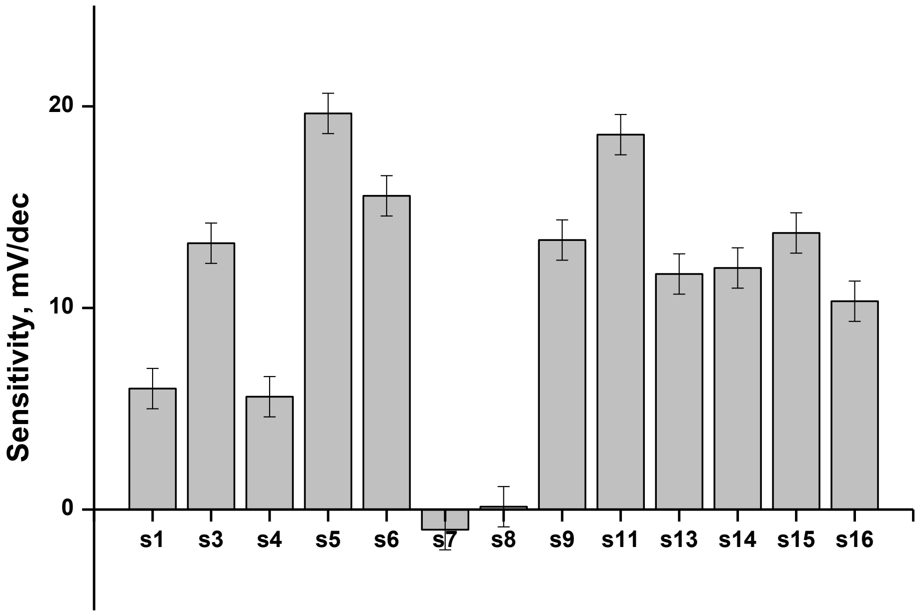

Figure S2. Sensitivity to Pu^{4+} in the presence of 500 mg/L uranium.

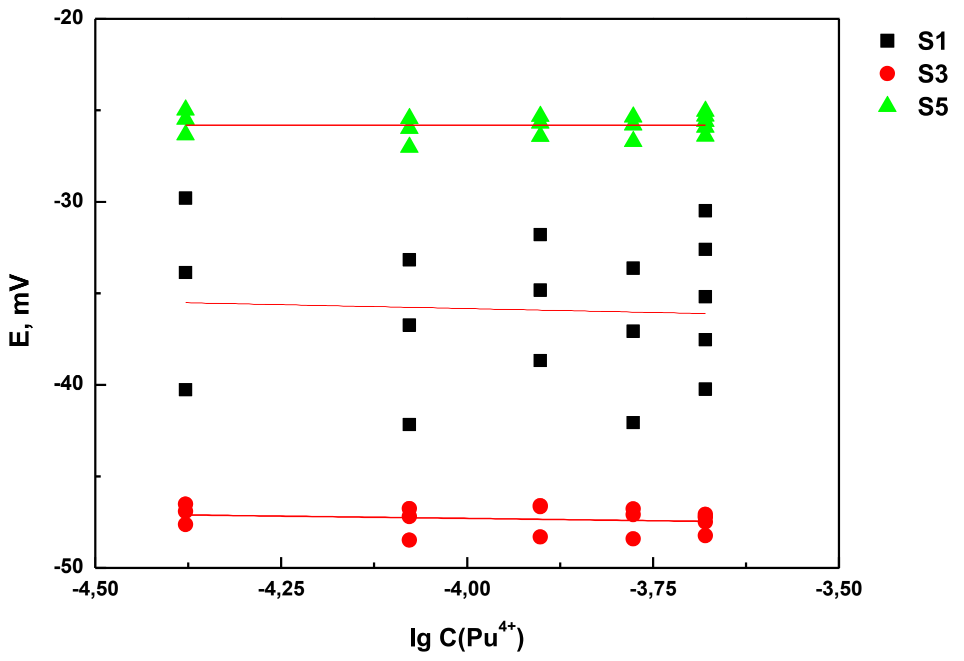

Figure S3. Sensor responses in the simulated PUREX solutions.

References:

[s1] M.Y. Alyapyshev, V.A. Babain, N.G. Antonov, I.V. Smirnov, Extraction of americium and europium from perchloric acid solutions with N,N′-dialkyl-and N,N,N′,N′-tetraalkylpyridine-2,6-dicarboxamides, Russ J Appl Chem+ 79(11) (2006) 1808-1815.

[s2] M.Y. Alyapyshev, V.A. Babain, L.I. Tkachenko, I.I. Eliseev, A.V. Didenko, M.L. Petrov, Dependence of Extraction Properties of 2,6-Dicarboxypyridine Diamides on Extractant Structure, Solvent Extraction and Ion Exchange 29(4) (2011) 619-636.

[s3] A. Shimada, T. Yaita, H. Narita, S. Tachimori, K. Okuno, Extraction Studies of Lanthanide(III) Ions with N,N′-Dimethyl-N,N′-diphenylpyridine-2,6-dicarboxyamide (DMDPhPDA) from Nitric Acid Solutions, Solvent Extraction and Ion Exchange 22(2) (2004) 147-161.

[s4] Y. Wen, Z. Qin, W. Liu, Extraction of Americium(III) and Europium(III) with Two Open-Chain Crown Ethers of Amide Type, Journal of Radioanalytical and Nuclear Chemistry 250(2) (2001) 285-289.

[s5] A.N. Turanov, V.K. Karandashev, N.I. Rodygina, A.M. Fedoseev, Extraction of rare-earth elements from nitric acid solutions with polyalkylphosphonitrilic acid, Radiochemistry 49(3) (2007) 264-267.

[s6] B.F. Myasoedov, M.K. Chmutova, N.E. Kochetkova, O.E. Koiro, G.A. Pribylova, N.P. Neeterova, T.Y. Medved, M.I. Kabachnik, [DIALKYLCARBAMOYLMETHYL] PHOSPHINE OXIDES ON THEIR EXTRACTION CAPACITY AND SELECTIVITY, Solvent Extraction and Ion Exchange 4(1) (1986) 61-81.

[s7] M. Alyapyshev, J. Ashina, D. Dar'in, E. Kenf, D. Kirsanov, L. Tkachenko, A. Legin, G. Starova, V. Babain, 1,10-Phenanthroline-2,9-dicarboxamides as ligands for separation and sensing of hazardous metals, RSC Advances 6(73) (2016) 68642-68652.

[s8] Y. Sasaki, Y. Tsubata, Y. Kitatsuji, Y. Sugo, N. Shirasu, Y. Morita, T. Kimura, Extraction Behavior of Metal Ions by TODGA, DOODA, MIDOA, and NTAamide Extractants from HNO3 to n-Dodecane, Solvent Extraction and Ion Exchange 31(4) (2013) 401-415.

[s9] M.Y. Alyapyshev, V.A. Babain, L.I. Tkachenko, A. Paulenova, A.A. Popova, N.E. Borisova, New Diamides of 2,2′-dipyridyl-6,6′-dicarboxylic Acid for Actinide-Lanthanide Separation, Solvent Extraction and Ion Exchange 32(2) (2014) 138-152.

[s10] L. N. Lazarev, M. F. Pushlenkov, V. A. Babain, V. M. Esimantovskii, V. A. Starchenko, E. G. Dzekun,; M. V. Gladyshev, V. M. Shidlovskii, Yu. Z. Prokopchuk. Russian Patent SU 1 589 858 A1, July 30 (1994).

[s11] M.Y. Alyapyshev, V.A. Babain, V.I. Boyko, I.I. Eliseev, D.O. Kirsanov, O.V. Klimchuk, A.V. Legin, E.S. Mikhailina, R.V. Rodik, I.V. Smirnov, Calixarenes functionalized with phosphine oxide and diamide functions as extractants and ionofores for rare-earth metals, J Incl Phenom Macro 67(1) (2010) 117-126.
